# Screening of *in vitro* antimicrobial effects of *Helicteres isora* extract against *Staphylococcus aureus*

**DOI:** 10.14202/vetworld.2021.2313-2316

**Published:** 2021-09-04

**Authors:** Sunisa Sirimongkolvorakul, Anusorn Jasancheun

**Affiliations:** 1 Department of Pre-Clinical Veterinary Science, Faculty of Veterinary Medicine, Mahanakorn University of Technology, 140 Cheum-Sampan Rd., Nong Chock, Bangkok 10530 Thailand; 2 A.T. Animal Clinic 284/3 Village No. 13, Kaset Wisai Roi Et, Thailand.

**Keywords:** antimicrobial, *Helicteres isora*, herbs, mastitis, *Staphylococcus aureus*

## Abstract

**Background and Aim:**

*Staphylococcus aureus* is an important pathogen causing contagious mastitis in cows that need novel treatment rather than antibiotic therapy. This study aimed to investigate the antimicrobial activity of *Helicteres isora* extracts against *S. aureus* isolated from subclinical and clinical mastitis cows using an *in vitro* model.

**Materials and Methods:**

*H. isora* pods were extracted using the following solvents: Distilled water, ethanol, acetone, and methanol. The antimicrobial activity of each extract was determined by the disk diffusion method and broth microdilution assay to assess the minimum inhibitory concentration (MIC) and minimum bactericidal concentration (MBC).

**Results:**

The ethanolic extract of *H. isora* exhibited the largest inhibition zones against *S. aureus* (31.05±1.20 mm), followed by the aqueous, methanolic, and acetone extracts, respectively (26.34±1.15, 24.23±0.50, and 22.46±1.53). The ethanolic extract also had a strong inhibitory effect on *S. aureus*, with MIC and MBC of 0.13 and 0.52 mg/mL, respectively.

**Conclusion:**

This study revealed that *H. isora* is a potential alternative natural antibacterial agent against *S. aureus* infection. The antimicrobial activity of *H. isora* is most likely mediated by phytochemical constituents.

## Introduction

Mastitis is economically important in dairy cows worldwide. Mastitis affects animal health, reduces milk quality, decreases milk yield, and shortens the productive life of affected cows [1,2]. Based on pathogens, mastitis can be divided into contagious and environmental pathogens [3]. *Staphylococcus aureus* is an important pathogen causing contagious mastitis characterized by lower cure rates than other pathogens [4,5]. The treatment of mastitis includes antibiotic use. However, the inappropriate use of antibiotics has led to many forms of bacterial resistance.

Research has focused on the potential of natural products in the treatment of mastitis, particularly *S. aureus* infection. Among herbs, *Helicteres isora* L. has shown medicinal importance. *H. isora* is also known as the Indian screw tree. It demonstrates various health benefits, including anticancer activity [6,7], antibacterial and antiplasmid activities [8], and cardiac antioxidant and antiperoxidative potency [9].

Therefore, this study aimed to investigate the antimicrobial activity of *H. isora* extract against *S. aureus* isolated from subclinical and clinical mastitis cows using an *in vitro* model. Furthermore, the active *H. isora* extracts were investigated for their phytochemical contents.

## Materials and Methods

### Ethical approval

Ethical approval was not necessary for this study as the milk samples were collected from lactating dairy cows from a private farm as per standard collection procedure.

### Study period and location

This study was conducted in April 2018. Milk samples were obtained from lactating cows from a private farm, Chachoengsao, Thailand. Then *S. aureus* was isolated and identified at Veterinary Diagnostic Center, Faculty of Veterinary Medicine, Mahanakorn University of Technology.

### Microbial sample collection

*S. aureus* was isolated from subclinical and clinical mastitis cows in Chachoengsao, Thailand. Isolates were identified by pure culture and biochemical tests. *S. aureus* ATCC II 25923 was used as a standard strain throughout the study.

### Preparation of extracts

*H. isora* pods were collected from Chachoengsao, Thailand. The herbs were identified by faculty member, Faculty of Pharmacy, Mahidol University, Bangkok, Thailand (Voucher no.PBM05249). Whole pods were washed several times with tap water, shade-dried (Thelco^®^, GCA/Precision Scientific, USA) at 45°C for 72 h, and ground into powder using a mixer blender (Otto, Thailand). Powdered *H. isora* (8 g) was soaked in distilled water, ethanol, acetone, and methanol. The plant material was left to stand at 37°C for 72 h and subsequently filtered through Whatman No. 1. The filtered extract was dried for 24 h using a freeze dryer (Supermodulyo-230, Thermo Scientific, USA).

### Determination of phytochemical constituents in *H. isora* extracts

The total phenolic content was determined by Folin–Ciocalteu assay [10] using gallic acid (GA) as a standard and expressed as mg/g GA equivalents (GAEs). Briefly, 0.5 mL extract (2.5 mg/mL) was mixed with 2.5 mL of 10% Folin–Ciocalteu reagent and 2 mL of 0.7 M sodium carbonate. The mixture was allowed to stand for 2 h at 37ºC (room temperature), and absorbance was measured by a spectrophotometer at 765 nm against distilled water as a blank. The results were expressed as mg/g GAE. All samples were analyzed in triplicate.

Flavonoid contents were determined based on the colorimetric aluminum chloride (AlCl_3_) method [10]. Briefly, 0.5 mL of extract was mixed with 0.1 mL of 10% AlCl_3_, 0.1 mL of 1 M potassium acetate, and 2.8 mL distilled water and left at room temperature for 30 min. The absorbance of the reaction mixture was measured at 415 nm. Total flavonoid contents were calculated as quercetin from a calibration curve. Quercetin in methanol was used for calibration curve preparation.

Tannin contents were determined based on a colorimetric assay [11]. Briefly, 2 mL extract was mixed with 2 mL of 0.1 M FeCl_3_ in 0.1 N HCl and 0.008 M potassium ferrocyanide. Absorbance was measured by a spectrophotometer at 120 nm. The results were expressed as mg/g GAE. All samples were analyzed in triplicate.

Alkaloid contents were determined based on a colorimetric method [11]. Briefly, 2 mL extract was mixed with 20% acetic acid and evaporated using a water bath. Concentrated ammonium was added dropwise to the extract until precipitation was complete.

### Determination of antimicrobial activity

The disk diffusion method was used for the primary screening of antibacterial activity of each extract [12]. A 10 ml (133 mg/mL) aliquot of each extract was individually applied to a sterile filter paper disk (Whatman No. 1; 8 mm in diameter). The disks were placed on Mueller-Hinton agar (MHA) plates previously seeded with the test bacteria [10^8^ colony-forming units (CFU)/mL]. Antibiotic susceptibility disk (5 mL ampicillin/disk) was used as a control, whereas 10 mL dimethyl sulfoxide (DMSO) was included as a negative control.

The plates were incubated at 37°C for 24 h. Antibacterial activity was evaluated by measuring the diameter (mm) of the inhibition zone. All tests were conducted in triplicates, and the mean diameter of the inhibition zone was calculated.

The minimum inhibitory concentration (MIC) and minimum bactericidal concentration (MBC) were determined by a modified broth microdilution assay [13]. The stock solution of each extract was prepared in DMSO. Two-fold serial dilutions of the extract were filtered through 0.45 mm Millipore filters and prepared at test concentrations from 66.5 to 0.13 mg/mL with an inoculum size of 1.5 × 10^7^ CFU/mL *S. aureus*. The lowest concentration of the extracts that showed no visible growth after 24 h incubation at 37°C was considered MIC. The positive growth of microorganisms cultured in Mueller Hinton Broth without test extracts served as a control. An aliquot of the mixture of the test extracts and bacterial suspension without turbidity after 24 h incubation was inoculated on MHA. To adjust the interference by plant pigments, a series of mixtures with uninoculated broth were prepared. The lowest concentration of the test extracts that showed no growth on agar was defined as MBC. Triplicate samples were performed for each concentration.

### Statistical analysis

Data were calculated as the mean±standard deviation. Analysis of variance was performed for each group. The significance of differences was considered when p≤0.05.

## Results

### Antimicrobial assay

The possibility of *H. isora* as an alternative antibacterial agent was tested. All test extracts of *H. isora* showed antimicrobial activity, inhibiting the growth of *S. aureus*. The ethanolic extract of *H. isora* exhibited the largest inhibition zones against *S. aureus* (31.05±1.20 mm), followed by the aqueous, methanolic, and acetone extracts, respectively (26.34±1.15, 24.23±0.50, and 22.46±1.53).

The efficacy of *H. isora* extract against *S. aureus* was investigated in terms of MIC and MBC using a broth microdilution assay. Table-1 shows the MIC values of each extracted sample. The ethanolic extract also had a strong inhibitory effect on *S. aureus*, with MIC and MBC of 0.13 and 0.52 mg/mL, respectively. Furthermore, the standard strain was more sensitive to *H. isora* than the clinical isolates. Moreover, the ethanolic extract of *H. isora* exhibited the highest concentration of phenolics and flavonoids, followed by the methanolic, acetone, and aqueous extracts. In contrast, the highest concentration of alkaloids and tannins was found in the aqueous extract. These findings indicated that the active compounds of *H. isora* extracts are different depending on the solvent used.

**Table 1:** MIC and MBC of H. isora extract against *S. aureus.*

Type of solvent extract	Isolated *S. aureus*		*S. aureus* ATCC II 25923	
	
	MIC (mg/mL)	MBC (mg/mL)	MIC (mg/mL)	MBC (mg/mL)
Methanol	33.25	>66.5	16.63	33.25
Ethanol	0.26	0.52	0.13	0.52
Acetone	>66.5	>66.5	>66.5	>66.5
Water	0.52	2.08	0.26	1.04

MIC=Minimum inhibitory concentration, MBC=Minimum bactericidal concentration, *H. isora=Helicteres isora,*

*S. aureus=Staphylococcus aureus*

### Phytochemical analysis

The preliminary phytochemical screening of each extract revealed a good source of tannins, phenolics, alkaloids, and flavonoids, and their quantities showed solvent-dependent variation (Table-2).

**Table 2:** Phytochemical presence of *H. isora* extract.

Type of solvent extract	Phytochemicals			

	Phenolics^1^ (mean±SE)	Flavonoids^2^ (mean±SE)	Tannins^1^ (mean±SE)	Alkaloids^3^ (mean± SE)
Methanol	36.52±1.5	32.25±1.1	1.02±0.5	1.60±0.1
Ethanol	42.16±1.3	25.17±0.8	1.32±0.3	1.8±0.5
Acetone	17.30±0.8	12.56±0.5	0.94±0.1	1.09±0.4
Water	28.71±1.1	16.30±1.2	2.80±0.2	3.2±0.2

Values were performed in triplicated and represented as mean±SE. ^1^mg GAE/100 g extract, ^2^mg QUE /100 g extract, ^3^mg DE/100 g extract. *H. isora=Helicteres isora*, GAE=Gallic acid equivalent

## Discussion

Various herbs are studied for their potential antimicrobial effects against pathogens, including *H. isora*. This study showed that the ethanol extract of *H. isora* and its constituents could be effective against *S. aureus*. *H. isora* extracts showed clear inhibition zones against *S. aureus* in the disk diffusion method. Furthermore, MIC and MBC demonstrated that *H. isora* extracts possessed antibacterial activity against clinic isolated bovine mastitis *S. aureus*, and their mechanism remains unknown. Notably, the ethanolic extract of *H. isora* exhibited the highest phenolics and flavonoids, whereas the aqueous extract of *H. isora* displayed the highest alkaloids and tannins. Thus, the antimicrobial properties of *H. isora* extracts may be due to the presence of flavonoids and other compounds. This finding was similar to several studies that reported that active compounds in peels, fruits, or seeds in herbs, such as phenolics, flavonoids, and tannins, exhibit antimicrobial activity. Indeed, flavonoids have shown antimicrobial effects against a wide range of organisms. Flavonoids have strong antimicrobial activity due to the structure-function relationship of flavonoids, thereby inhibiting the growth of microorganisms [14]. The ethanolic extract of grape pomace had antimicrobial activity against Enterobacteriaceae, *S. aureus*, *Salmonella*, yeast, and molds [15]. Mango kernel extracts exhibited antimicrobial spectrum against Gram-positive and Gram-negative bacteria, such as *S. aureus*, *Bacillus subtilis*, and *Escherichia coli* [16]. Similarly, the phenolic extracts of honey had strong antimicrobial activity against *E. coli*, followed by *S. aureus*, *Enterococcus faecalis*, and *Pseudomonas aeruginosa* [12]. Studies that investigated the antimicrobial activity of various herbs against *S. aureus* have also been reported. The essential oil of *Eucalyptus globulus* leaves had antimicrobial effects as determined by disk diffusion and dilution broth methods [17]. Similarly, cinnamon essential oil exhibited antibacterial activity against *S. aureus* (MIC=1.0 mg/mL), and the mechanism changes membrane permeability and integrity [18]. Honey from different botanical origins also had different MICs against *S. aureus* [19]. *Litsea cubeba* essential oil showed good antibacterial effects against methicillin-resistant *S. aureus* (MRSA) by destructing the MRSA cell membrane, leading to intracellular biological macromolecular leakages [20]. Nevertheless, several studies demonstrated that the antimicrobial activity of herbs could vary depending on the extraction method, inoculum size, and determination method [21].

## Conclusion

This study revealed that *H. isora* is a potential alternative natural antibacterial agent against *S. aureus* infection. The antimicrobial activity of *H. isora* is most likely mediated by phytochemical constituents.

## Authors’ Contributions

SS and AJ: Designed and managed the study. AJ: Performed sample collections. SS and AJ: Data collection and analysis. SS: Drafted the manuscript. All authors participated in the revision of this manuscript and approved the submission.
